# PReVENT - protective ventilation in patients without ARDS at start of ventilation: study protocol for a randomized controlled trial

**DOI:** 10.1186/s13063-015-0759-1

**Published:** 2015-05-24

**Authors:** Fabienne D. Simonis, Jan M. Binnekade, Annemarije Braber, Harry P. Gelissen, Jeroen Heidt, Janneke Horn, Gerard Innemee, Evert de Jonge, Nicole P. Juffermans, Peter E. Spronk, Lotte M. Steuten, Pieter Roel Tuinman, Marijn Vriends, Gwendolyn de Vreede, Rob B. de Wilde, Ary Serpa Neto, Marcelo Gama de Abreu, Paolo Pelosi, Marcus J. Schultz

**Affiliations:** Department of Intensive Care & Laboratory of Experimental Intensive Care and Anesthesiology, Academic Medical Center, University of Amsterdam, Meibergdreef 9, 1105 AZ Amsterdam, The Netherlands; Department of Intensive Care, Gelre Hospitals, Apeldoorn, The Netherlands; Department of Intensive Care, Tergooi, Hilversum, The Netherlands; Department of Intensive Care, Leiden University Medical Center, Leiden, The Netherlands; Department of Health Technology and Services Research, Twente University, Enschede, The Netherlands; Department of Intensive Care & REVIVE Research VUmc Intensive Care, VU Medical Center, Amsterdam, The Netherlands; Department of Critical Care Medicine, Hospital Israelita Albert Einstein, São Paulo, Brazil; Department of Anesthesiology and Intensive Care, University Hospital Carl Gustav Carus, Dresden, Germany; Department of Surgical Sciences and Integrated Diagnostics, IRCCS San Martino IST, University of Genoa, Genoa, Italy

**Keywords:** Mechanical ventilation, Ventilator-induced lung injury, Tidal volume, Respiratory rate, Protective ventilation, Intensive care unit, Critical care, Non injured lungs

## Abstract

**Background:**

It is uncertain whether lung-protective mechanical ventilation using low tidal volumes should be used in all critically ill patients, irrespective of the presence of the acute respiratory distress syndrome (ARDS). A low tidal volume strategy includes use of higher respiratory rates, which could be associated with increased sedation needs, a higher incidence of delirium, and an increased risk of patient-ventilator asynchrony and ICU-acquired weakness. Another alleged side-effect of low tidal volume ventilation is the risk of atelectasis. All of these could offset the beneficial effects of low tidal volume ventilation as found in patients with ARDS.

**Methods/Design:**

PReVENT is a national multicenter randomized controlled trial in invasively ventilated ICU patients without ARDS with an anticipated duration of ventilation of longer than 24 hours in 5 ICUs in The Netherlands. Consecutive patients are randomly assigned to a low tidal volume strategy using tidal volumes from 4 to 6 ml/kg predicted body weight (PBW) or a high tidal volume ventilation strategy using tidal volumes from 8 to 10 ml/kg PBW. The primary endpoint is the number of ventilator-free days and alive at day 28. Secondary endpoints include ICU and hospital length of stay (LOS), ICU and hospital mortality, the incidence of pulmonary complications, including ARDS, pneumonia, atelectasis, and pneumothorax, the cumulative use and duration of sedatives and neuromuscular blocking agents, incidence of ICU delirium, and the need for decreasing of instrumental dead space.

**Discussion:**

PReVENT is the first randomized controlled trial comparing a low tidal volume strategy with a high tidal volume strategy, in patients without ARDS at onset of ventilation, that recruits a sufficient number of patients to test the hypothesis that a low tidal volume strategy benefits patients without ARDS with regard to a clinically relevant endpoint.

**Trial registration:**

The trial is registered at www.clinicaltrials.gov under reference number NCT02153294 on 23 May 2014.

## Background

Mechanical ventilation is generally seen as an invasive but foremost safe supportive strategy in critically ill patients. However, there is unequivocal and increasing evidence from both experimental and clinical studies that ventilation has a strong potential to aggravate, or even initiate injury to lungs [1, 2] and respiratory muscles [3, 4]. Indeed, mechanical ventilation may result in a ventilation pattern of ventral overstretching and dorsal collapse of lung tissue, which both play a role in development of so-called ‘ventilator-induced lung injury’ (VILI). Mechanical ventilation is also associated with respiratory muscle disuse and misuse, with concomitant atrophy of diaphragmatic myofibers that plays a role in development of so-called ‘ventilator-induced diaphragm dysfunction’ (VIDD) [5].

The harmful effects of the traditional use of high tidal volumes were not recognized until 2000 when the beneficial effects of ventilation using a low tidal volume strategy (6 ml/kg predicted body weight, PBW) in patients with the acute respiratory distress syndrome (ARDS) were established in the landmark NHLBI ARDS Network trial [6]. Some clinicians and investigators were reluctant to accept these findings, but subsequent trials and a meta-analysis convincingly confirmed the reduction in mortality by using these lung-protective ventilator settings in patients with ARDS [7]. Currently, lung-protective ventilation with low tidal volumes is considered standard of care for patients with ARDS [8–10]. It is uncertain, however, whether we should use a low tidal volume strategy in all ICU patients: that is irrespective of the presence of ARDS. While the results of a recent meta-analysis suggests that tidal volume reduction also benefits patients without ARDS [11], we should realize that the meta-analyzed studies all had methodological shortcomings and the majority of them focused on ventilation during general anesthesia for surgery, which cannot be simply generalized to other clinical situations like ventilation of critically ill patients in the ICU setting.

Numerous arguments against indiscriminate use of low tidal volume strategies have been raised. One argument is that this strategy necessitates higher respiratory rates, which could increase sedation needs [12], risk of ICU delirium [13] or ICU-acquired weakness [14]. Other arguments against unselective use of lower tidal volumes are that it could promote collapse of lung tissue [15], and increases the risk of patient-ventilator asynchrony [16, 17]. In addition, the increased efforts of patients on spontaneous ventilation using lower tidal volumes could induce more so-called *pendelluft*, thereby actually increasing the risk of lung injury [18]. Finally, another alleged side-effect of higher respiratory rates is patient fatigue, although there are no studies that support this fear.

The abovementioned suggested side-effects of low tidal volume ventilation strategies could offset its potential beneficial effects, especially in patients without ARDS in whom the benefits of using low tidal volumes could be less than in those with ARDS. Consequently, lung-protective ventilation using low tidal volumes is not yet recommended in guidelines for ventilation of patients without ARDS, resulting in remarkable and unwanted practice variation [19, 20]. The ICU community explicitly requests a well-powered trial comparing ventilation with low tidal volumes to ventilation with high tidal volumes in patients without ARDS [2, 11, 21, 22], a trial that should use patient-relevant and objective outcomes, including duration of ventilation and ICU and hospital length of stay (LOS).

The objective of the present trial, therefore, is to determine whether ventilation with tidal volumes from 6 down to 4 ml/kg PBW, as compared to ventilation with tidal volumes from 8 up to 10 ml/kg PBW reduces duration of mechanical ventilation in ICU patients without ARDS at onset of ventilation. Specifically, we hypothesize that ventilation with low tidal volumes increases the number of days alive and free from ventilation at day 28.

## Methods

### Design

The PReVENT (PRotective VENTilation in patients without ARDS at start of ventilation) trial is an investigator-initiated, national, multicenter, parallel randomized controlled two-arm trial in intubated and ventilated ICU patients not suffering from ARDS at onset of ventilation. The PReVENT trial will be conducted according to the principles of the *Declaration of Helsinki* as stated in the current version of Fortaleza, Brazil, 2013 [23] and in accordance with the Medical Research Involving Human Subjects Act (WMO). The Institutional Review Board of the Academic Medical Center, Amsterdam, The Netherlands, approved the trial protocol under reference number 2014_075#B2014424. The trial is registered at www.clinicaltrials.gov (NCT02153294). Patients will be provisionally included under a strategy of deferred consent (see below).

### CONSORT diagram

**Fig. 1:**
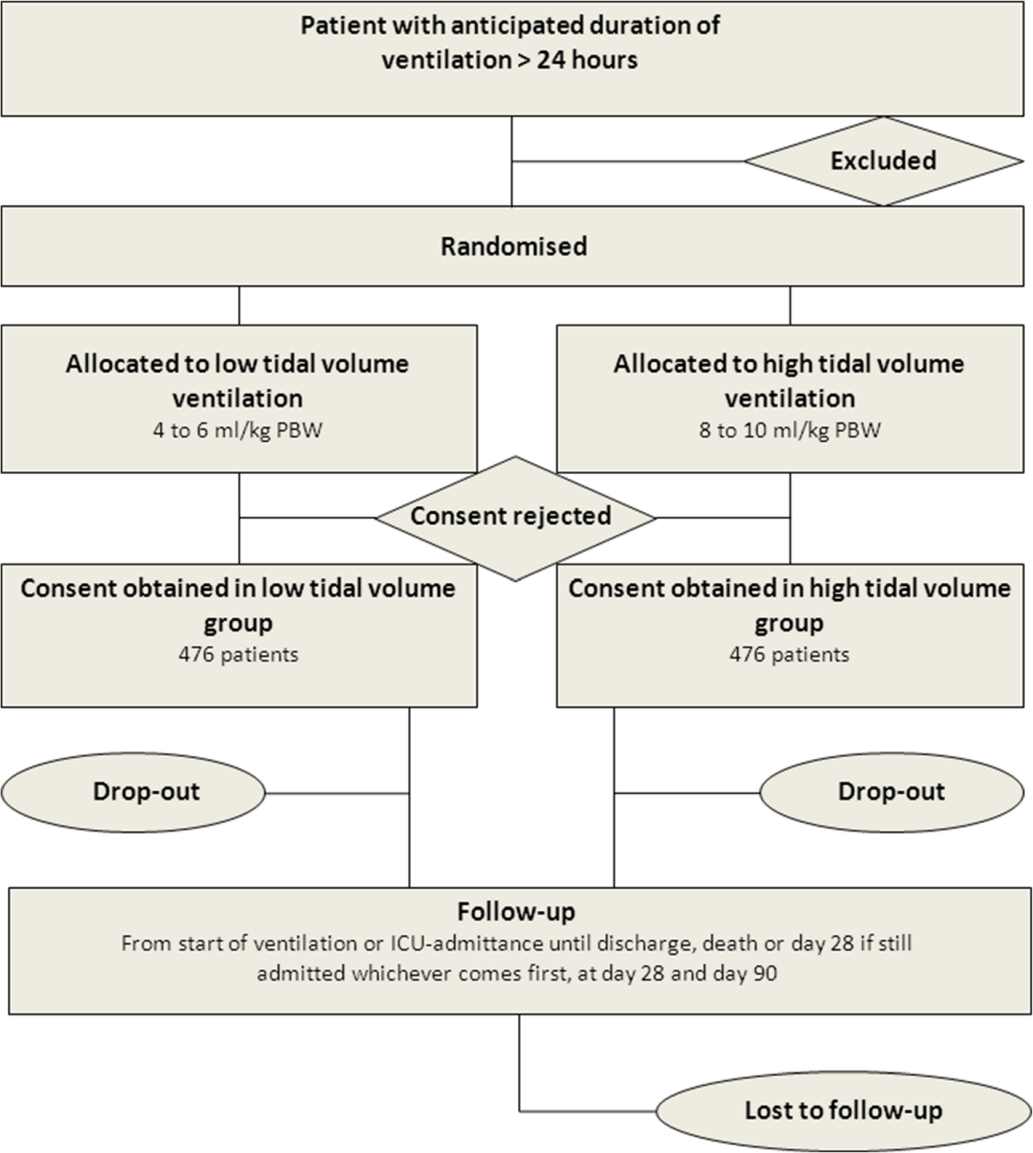
Consolidated Standards of Reporting Trials (CONSORT) diagram. PBW = predicted body weight

The Consolidated Standards of Reporting Trials (CONSORT) [24] diagram of PReVENT is presented in Fig. 1. Consecutive patients admitted to one of the participating ICUs who need invasive ventilation are screened. Demographic data are registered regardless of meeting enrollment criteria. If excluded from participation, the reason(s) for exclusion are registered.

### Setting

PReVENT is performed in the ICUs of five centers in The Netherlands: three university centers (Leiden University Medical Center in Leiden, VU Medical Center in Amsterdam and Academic Medical Center in Amsterdam), and two teaching hospitals (Gelre Ziekenhuizen in Apeldoorn and Tergooi in Hilversum).

### Study population

Intubated and ventilated ICU patients are eligible for participation if the need of ventilator support for longer than 24 hours is expected. Notably, patients who already received ventilation before ICU admission: for example, in the emergency room or in the operation room, are also eligible, but need to be included within 1 hour of ventilation in the ICU. Patients suspected of having ARDS at start of ventilation, according to the Berlin definition [25], must be excluded from participation. The PReVENT trial accepts patients with a PaO_2_/FiO_2_ between 200 and 300 mmHg, but excludes patients with a PaO_2_/FiO_2_ < 200 mmHg within the first hour of ventilation unless it is likely that fluid overload or cardiac failure are the cause of hypoxia. Non-adult patients (age < 18 years) are excluded, as are patients previously randomized in PReVENT or participating in other interventional trials, any session of ventilation longer than 12 hours directly preceding current ICU admission, pregnant patients, patients with increased and uncontrollable intracranial pressure (of ≥ 18 mmHg), patients suffering from GOLD classification III or IV chronic obstructive pulmonary disease (COPD), with status asthmaticus or premorbid restrictive pulmonary disease (evidence of chronic interstitial infiltration on previous chest radiographs), also patients with new proven pulmonary thrombo-embolism or any previous pneumectomy or lobectomy. Informed consent will be obtained from all participants or a legal representative in case the former is impossible.

### Randomization and blinding

Randomization will be performed using a dedicated, password protected, SSL-encrypted website with ALEA® software (TenALEA consortium, Amsterdam, The Netherlands) using random block sizes and is stratified only per ICU and per intubation location (that is intubated in the ICU or before ICU admittance in the emergency room or operation room). When, after randomization, consent is rejected, the patient is excluded. Due to the nature of the intervention, blinding is not possible.

### The ventilation strategies to be compared

In both arms the size of tidal volumes is titrated on the PBW, which is calculated according to a previously used formula [6]:

$$ \begin{array}{l}\mathsf{50} + \mathsf{0.91}\ \mathit{\mathsf{x}}\ \left( centimeters\  of\  height\ \hbox{--}\ \mathsf{152.4}\right)\ for\  male,\  and\\ {}\mathsf{45.5} + \mathsf{0.91}\ \mathit{\mathsf{x}}\ \left( centimeters\  of\  height\ \hbox{--}\ \mathsf{152.4}\right)\ for\  females\end{array} $$

Patients randomized to the ‘low tidal volume’-arm start with a tidal volume of 6 ml/kg PBW. The tidal volume size is decreased in steps of 1 ml/kg PBW per hour, to a minimum of 4 ml/kg PBW, unless the patient suffers from severe dyspnea (identified by increased respiratory rate > 35 breaths per minute accompanied by increasing levels of discomfort with or without need for more sedation) or unacceptable acidosis (Table 1). The following actions could be taken to prevent respiratory acidosis: increasing respiratory rate, removal of the heat and moisture exchanger and decreasing instrumental dead space by shortening ventilation tubing, to limit dead space ventilation. Patients randomized to the low tidal volume arm may need very little support when the ventilator is switched to pressure support ventilation, but a minimum of 5 cmH_2_O should be used. In case the resulting tidal volume exceeds 6 ml/kg PBW this must be accepted (that is this is neither a reason to use (more) sedation and/or muscle relaxants, nor to switch to volume-controlled ventilation) (see weaning).

**Table 1:** Ventilator settings with the two ventilation strategies

	Lower tidal volume ventilation	Higher tidal volume ventilation
Ventilator mode	Assisted MV = volume- controlled	Assisted MV = volume-controlled
	Spontaneous MV = pressure support	Spontaneous MV = pressure support
Target tidal volume	4 ml/kg PBW	10 ml/kg PBW
Allowable tidal volume in case of high pressures (in the high tidal group) or severe dyspnea (in the low tidal group)	6 ml/kg PBW^a^	8 ml/kg PBW^a^
Allowable ventilator rate setting needed to achieve normal pH (7.25 to 7.45)	6 to 35 breaths/minute; typically higher than with an higher tidal volume	6 to 35 breaths/minute; typically lower than with a lower tidal volume
Inspiration-to-expiration ratio	1:2	1:2
PaO_2_ or SpO_2-_targets	7.3 to 10.7 kPa (55 to 80 mmHg), or 90 to 92 %	7.3 to 10.7 kPa (55 to 80 mmHg), or 90 to 92 %
Allowable combinations of FiO_2_ and PEEP	0.21 and 5 cmH_2_O	0.21 and 5 cmH_2_O
	0.30 and 5 cmH_2_O	0.30 and 5 cmH_2_O
	0.40 and 5 cmH_2_O	0.40 and 5 cmH_2_O
	0.40 and 8 cmH_2_O	0.40 and 8 cmH_2_O
	0.50 and 8 cmH_2_O	0.50 and 8 cmH_2_O
	0.50 and 10 cmH_2_O	0.50 and 10 cmH_2_O
	0.60 and 10 cmH_2_O	0.60 and 10 cmH_2_O
Daily assessment of the ability to breathe with pressure support	Required when FiO_2_ ≤ 0.4 or earlier	Required when FiO_2_ ≤ 0.4 or earlier
Allowable tidal volume when ARDS develops	6 ml/kg PBW	6 ml/kg PBW
Allowable tidal volume when ARDS develops; in case of severe dyspnea or unacceptable pH	8 ml/kg PBW	8 ml/kg PBW
Allowable combinations of FiO_2_ and PEEP (continued)	0.21 and 5 cmH_2_O	0.21 and 5 cmH_2_O
	0.30 and 5 cmH_2_O	0.30 and 5 cmH_2_O
	0.40 and 5 cmH_2_O	0.40 and 5 cmH_2_O
	0.40 and 8 cmH_2_O	0.40 and 8 cmH_2_O
	0.50 and 8 cmH_2_O	0.50 and 8 cmH_2_O
	0.50 and 10 cmH_2_O	0.50 and 10 cmH_2_O
	0.60 and 10 cmH_2_O	0.60 and 10 cmH_2_O
	0.70 and 10 cmH_2_O	0.70 and 10 cmH_2_O
	0.70 and 12 cmH_2_O	0.70 and 12 cmH_2_O
	0.70 and 14 cmH_2_O	0.70 and 14 cmH_2_O
	0.80 and 14 cmH_2_O	0.80 and 14 cmH_2_O
	0.90 and 14 cmH_2_O	0.90 and 14 cmH_2_O
	0.90 and 16 cmH_2_O	0.90 and 16 cmH_2_O
	0.90 and 18 cmH_2_O	0.90 and 18 cmH_2_O
	1 and 18 cmH_2_O	1 and 18 cmH_2_O
	1 and 20 cmH_2_O	1 and 20 cmH_2_O
	1 and 22 cmH_2_O	1 and 22 cmH_2_O
	1 and 24 cmH_2_O	1 and 24 cmH_2_O

^a^with pressure support tidal volumes could be higher than targeted with the lowest possible pressure support level, or lower than targeted with the highest allowed maximum airway pressure - this is accepted and never a reason for use of (more) sedation, paralytic agents, and/or switch to a controlled mode of ventilation

*ARDS* acute respiratory distress syndrome, *FiO*_*2*_ O_2_–fraction of inspired air, *MV* mechanical ventilation, *PBW* predicted body weight, *PEEP* positive end-expiratory pressure, *SpO*_*2*_ peripheral O_2_ saturation

Patients randomized to the ‘high tidal volume’-arm start with a tidal volume of 10 ml/kg PBW. With volume-controlled ventilation the plateau pressure should not exceed 25 cm H_2_O [2]. Only if the plateau pressure exceeds 25 cmH_2_O the tidal volume is decreased in steps of 1 ml/kg PBW per hour, to a minimum of 8 ml/kg PBW (Table 1). Patients randomized to the high tidal volume arm generally need more support when the ventilator is switched to pressure support ventilation, but the maximal airway pressure should never exceed 25 cmH_2_O [2]. In case the resulting tidal volume remains below 10 ml/kg PBW this is accepted (that is this is neither a reason to use (more) sedation and/or muscle relaxants, nor to switch the ventilator to volume-controlled ventilation) (see weaning).

### Other ventilator settings

The allowed ventilation modes are volume-controlled ventilation and pressure support ventilation. The inspiration-to-expiration ratio with volume-controlled ventilation is 1:2. With volume-controlled ventilation the inspiration time and pause are set at 25 % and 10 % respectively. With pressure support ventilation the highest possible pressure rise is chosen, and cycling off is set at 25 %. The inspired oxygen fraction is 0.21 or higher to maintain oxygen saturation 90 to 92 % and/or PaO_2_ > 7.3 to 10.7 kPa (55 to 80 mmHg). The respiratory rate is adjusted to maintain a blood pH of 7.25 to 7.45. In case of metabolic acidosis or - alkalosis, a lower or higher than normal PaCO_2_ can be accepted, left to the discretion of the attending physician. The lowest level of positive end-expiratory pressure (PEEP) is 5 cmH_2_O; allowed FiO_2_-PEEP-combinations are provided in Table 1. Recruitment maneuvers are allowed, when deemed necessary, left to the discretion of the attending physician.

### Weaning from ventilation

Daily assessment of the ability to breathe with pressure support ventilation is required as soon as FiO_2_ ≤ 0.4 or when the PEEP level and FiO_2_ level are lower than the day before. In addition, the ventilator can be switched to pressure support ventilation at any moment the attending nurse or physician considers the patient is awake enough to breathe with pressure support ventilation. Assessment of the ability to breathe with pressure support is also required in case patient-ventilator asynchrony is noticed (that is ineffective breathing; double triggering, use of accessory respiratory muscles).

A patient is assumed to be ready for extubation when the following criteria are met for at least 30 minutes, the final decision for extubation is made by the attending physician: a patient is responsive and cooperative, has an adequate cough reflex, PaO_2_/FiO_2_ of > 200 mmHg with FiO_2_ ≤ 40 % and a respiratory rate of 8 to 30/minute with no signs of respiratory distress (that is marked accessory muscle use, abdominal paradox, diaphoresis, marked dyspnea), the pressure support level is < 7 cmH_2_O (lower tidal volume arm) or < 12 cmH_2_O (higher tidal volume arm) with a temperature of > 36.0 °C and < 38.5 °C and is hemodynamically stable (systolic blood pressure 80 to 160 mmHg and heart rate 40 to 130/minute) with no uncontrolled arrhythmia.

In the high tidal volume arm physicians and nurses may lower the pressure support level first (that is before extubation), to see whether patients can ventilate at the lowest support level. This is not mandatory, though, as extubation is also allowed with higher pressures. For this, the pressure support level is lowered step-wise with steps of 2 to 5 cmH_2_O per hour to < 7 cmH_2_O. If this is not tolerated according to the conditions mentioned above, the pressure support level is set back to maintain a tidal volume as per randomization and the patient is assessed for extubation the following shift.

If a patient is taken off mechanical ventilation but subsequently requires additional ventilation within 28 days after randomization, the same tidal volume protocol is used. Non-invasive ventilation is allowed, but an attempt should be made to have comparable tidal volumes as with invasive ventilation, as per randomization.

### Tracheostomy

Early tracheostomy has no advantage over late tracheostomy [26]. Therefore, tracheostomy is only to be performed on strict indications and preferably not earlier than 10 days after intubation. Strict indications for tracheostomy are failure to intubate, expected duration of ventilation > 14 days, Glasgow Coma Score < 7 and/or inadequate swallow or cough reflex with retention of sputum, severe ICU-acquired weakness, prolonged or unsuccessful weaning and repeated respiratory failure after tracheal extubation.

### Standard procedures

Sedation follows the local guidelines in each participating hospital. In general, these guidelines favor the use of analgo-sedation over hypno-sedation, use of bolus over continuous infusion of sedating agents, and the use of sedation scores. Nurses determine the level of sedation at least three times per day. The adequacy of sedation in each patient is evaluated using a Richmond Agitation Sedation Scale (RASS) [27, 28]; a RASS score of −2 to 0 is considered adequate sedation. As stated above, sedation adjustments should never be done to allow a lower or higher tidal volume. The goals of sedation are to reduce agitation, stress and fear; to reduce oxygen consumption (heart rate, blood pressure and minute volume are measured continuously); and to reduce physical resistance to, and fear of, daily care and medical examination. Patient comfort is the primary goal. Level of pain is determined using Numeric Rating Scale (NRS), Visual Analog Scale (VAS), Critical Care Pain Observation Tool (CCPOT) or Behavioral Pain Scale (BPS).

To prevent nosocomial infections, selective oropharyngeal decontamination (SOD) or selective decontamination of the digestive tract (SDD) is performed in all patients who are expected to need ventilation for longer than 48 hours, and/or are expected to stay in ICU for longer than 72 hours [29].

Thrombosis prophylaxis is indicated for all patients who are not treated with anticoagulants: for example, for therapeutic reasons or systemic prophylaxis because of an implanted device or extra-corporeal circulation like veno-venous hemofiltration. Thrombosis prophylaxis will be given according to local guidelines.

A fluid balance targeted at normovolemia and a diuresis of ≥ 0.5 ml/kg/hour should be maintained. Crystalloid infusions are preferred over colloid infusions.

A hypo-caloric, protein-rich diet (1.2 to 1.7 g/kg bodyweight/24 hours) is started as soon as possible after ICU admission. Enteral nutrition with a feeding gastric tube is preferred over intravenous feeding. If stomach retention occurs a duodenal tube can be used if administration of prokinetics is not sufficient, according to local guidelines. When optimal protein intake cannot be reached within 4 days, additional parenteral nutrition can be started.

### Follow-up

On ICU admission and within the first 24 hours, demographic and baseline data, as well as data on disease severity are collected. Data collection includes: gender, age, height, weight, reason for ICU admission, reason for ventilation, cause of respiratory failure, the Acute Physiology and Chronic Health Evaluation II (APACHE II) score and the Simplified Acute Physiology Score II (SAPS II).

Data on standard of care and clinical outcome variables (described below) are collected on a daily basis every day until day 28, discharge of the ICU or death, whichever comes first. Data on duration of ventilation, length of stay in ICU and hospital, location of the patient (in ICU, hospital, other facility, or home) and life status (alive or deceased) are assessed on days 28 and 90.

The following variables are collected daily: respiratory status; intubation status (if extubated: time of extubation/if re-intubated: time of re-intubations) tracheostomy status (if tracheostomized: time of tracheotomy), invasiveness of ventilation (invasive, non-invasive, or intermittent ventilation via tracheostomy), need for decreasing instrumental dead space; development of pulmonary complications (moderate or severe ARDS, pneumonia, atelectasis and pneumothorax); cumulative use and duration of sedatives and neuromuscular blocking agents; sedation score using the RASS [27, 28]; level of pain (NRS or VAS or CCPOT or BPS); delirium score with the Confusion Assessment Method for ICU (CAM-ICU) score [30, 31]; presence of ICU-acquired weakness using Medical Research Council (MRC) score [32] or Grip Strength Assessment [33].

The following mechanical ventilation parameters are collected within 1 hour before and 1 hour after randomization, and every day at a fixed time point until cessation of ventilation: tidal volume in ml and ml/kg PBW, respiratory rate, level of PEEP, peak and plateau pressures, or level of pressure support and maximal airway pressure, inspiration to expiration ratio, inspired oxygen fraction, minute volume, pulmonary compliance, Lung Injury Score (LIS: based on chest X-ray findings, PaO_2_/FiO_2_, PEEP level and respiratory compliance) [34] and Oxygenation Index [35].

ICU-related therapy variables to collect daily include: respiratory parameters and arterial blood gas analysis (once daily), amount and type of infused products including blood products and fluids (crystalloids and (artificial) colloids), cumulative fluid balance and Sequential Organ Failure Assessment (SOFA) score [36].

Resource use parameters and unit prices are collected to estimate health care costs from a health systems perspective and include costs of ventilation, costs of stay in ICU, costs of stay in hospital, costs of cumulative use of sedatives, costs of neuromuscular blocking agents, costs of use of tracheostomies and costs of ventilator-associated pneumonia (VAP).

### Study endpoints

The primary endpoint is the number of ventilator-free days and alive at day 28, defined as the number of days from day 1 to day 28 the patient is alive and breathes without invasive assistance of the mechanical ventilator for at least 24 consecutive hours. To calculate this endpoint all relevant data will be taken into account and collected, including repeated re-intubation and -extubation.

Secondary endpoints are subdivided into clinical outcome variables and health-economic outcome variables. Clinical outcome variables include: ICU- and hospital length of stay (LOS), ICU-, hospital-, and 90-day mortality, development of ARDS, VAP, development of atelectasis and presence of pneumothorax and cumulative use and duration of use of sedatives, cumulative use and duration of use of neuromuscular blocking agents (other than used for intubation), and ICU-acquired weakness and delirium. Health-economic variables include incremental cost per ICU-day avoided and incremental cost per mechanical ventilation-day avoided.

### Statistical considerations

We will include a total of 952 patients. The required sample size is calculated using data from the recently published meta-analysis [11] and a secondary analysis of this meta-analysis using individual patient data from the studies performed in ICU patients [37]. The sample size is computed and based on the hypothesis that ventilation with low tidal volumes is associated with a reduction of 1 day of ventilation. A sample size of 397 patients in each group has 80 % statistical power to detect a difference of 1 ventilator-free day and alive at day 28 after ICU admission, assuming that the common standard deviation is 5 using a 2 -group *t-*test with a 0.05 2-sided significance level. The sample size is increased by 20 % to correct for dropouts and those lost to follow-up meaning that each group will contain 476 patients.

For this study we will include patients using a strategy using deferred informed consent because we explicitly want to randomize and start ventilation according to randomization within 1 hour after start of ventilation, or within 1 hour after admission if ventilation was initiated in the emergency room or the operation room. Nevertheless, written informed consent from the legal representative will be requested as soon as possible thereafter, and never later than 24 hours after randomization. If informed consent is not obtained within those 24 hours, or if a legal representative denies participation within this time frame, the patient is excluded and data will no longer be used, nor will this patient be counted for the sample size of 476 inclusions in each group (that is the provisionally included patient for whom informed consent is not obtained within the time frame of 24 hours is ‘replaced’ by a new patient, until the total number of 476 patients in each arm is definitively included).

### Statistical analysis

The primary outcome, the number of days alive and free of ventilation at day 28, will be analyzed using Cox’s regression. Possible imbalance between groups will be modeled in the Cox model. *P*-values of 0.05 are used for statistical significance. When appropriate, statistical uncertainty will be expressed by the 95 % confidence levels. All statistical analyses will be performed with the R language and environment for statistical computing.

Continuous normally distributed variables will be expressed by their mean and standard deviation or when not normally distributed as medians and their interquartile ranges. Categorical variables will be expressed as n (%). To test groups Student’s *t*-test will be used, if continuous data is not normally distributed the Mann-Whitney *U*-test will be used. Categorical variables will be compared with the Chi-square test or Fisher’s exact tests. Time dependent data will be analyzed using a proportional hazard model adjusted for possible imbalances of patients’ baseline characteristics. Analysis will be performed with R statistics version 3.0.2 (R Foundation, Vienna, Austria). Patient characteristics will be compared and described by appropriate statistics.

The goal of the primary analysis is to quantify the effect of ventilation using low tidal volumes versus ventilation using high tidal volumes on the number of ventilator-free days and alive at day 28. Statistical analysis will be based on the intention-to-treat principle. We will also perform a per-protocol analysis, comparing patients who received lower tidal volumes and patients who received higher tidal volumes. Other secondary analyses include*:* patients who had pneumonia versus patients without pneumonia, and patients with sepsis versus patients without sepsis.

### Study organization

The steering committee is composed of the principal investigator, the coordinating investigator, the local investigators in the participating ICUs, and six (inter)-national experts of ventilation who contribute to the design and revisions of the study protocol. The coordinating investigator is responsible for administrative management and communication with the local investigators and provides assistance to the participating clinical sites in trial management, record keeping and data management. The coordinating investigator helps in setting up local training in the participating ICUs to ensure the study is conducted according to the ICH-GCP guidelines, to guaranty integrity of data collection and to ensure timely completion of the case report forms. The local investigators provide structural and scientific leadership. They guarantee the integrity of data collection and ensure timely completion of the case report forms.

An independent monitor is installed to perform study monitoring. Remote monitoring by means of queries on the database will be done by a statistician and analyzed by the monitor to signalize early aberrant patterns, trends, issues with consistency or credibility and other anomalies. On-site monitoring will comprise controlling presence and completeness of the research dossier and the informed consent forms, source data checks will be performed in the files of 25 % of the patients. Each ICU will be visited at least once every year.

An independent Data and Safety Monitoring Board (DSMB) watches over the ethics of conducting the study in accordance with the *Declaration of Helsinki*, monitors safety parameters and the overall conduct of the study. The DSMB is composed of five independent individuals (Prof. Antonio Artigas Raventós, Prof. Thomas Bein, Prof. Ognjen Gajic, Dr. Diederik Gommers and Prof. Herman Wrigge). The DSMB will meet by conference calls. The first meeting is scheduled after the first 150 patients. Subsequent to this meeting the DSMB will meet every 6 months.

As this study compares two treatment strategies that are used in standard care, no related serious adverse events are expected. All unexpected adverse events will be reported to the DSMB. Any report and/or advice of the DSMB will be send to the sponsor of the study, the Academic Medical Center, Amsterdam, The Netherlands. Should the sponsor decide not to fully implement advices of the DSMB, the sponsor will send the advice to the reviewing Institutional Review Board, including a note to substantiate why (part of) the advice of the DSMB will not be followed.

## Discussion

PReVENT is the first randomized controlled trial that is sufficiently powered to test the hypothesis that a ventilation strategy using low tidal volumes, as compared to a ventilation strategy using high tidal volumes, benefits patients without ARDS with regard to the clinically relevant endpoint of duration of ventilation.

It is increasingly becoming clear that ventilation *per se* has the potential to initiate lung injury or ARDS, mainly due to over-distension of (parts of) the lung [2]. Since a generally employable alternative treatment for ventilation is not yet at our disposal, the prevention of over-distension and lung injury should be the target. Earlier clinical studies indicated that ventilation using low tidal volumes could prevent development of lung injury by avoiding excessive stretch, and that high tidal volumes are more likely to cause damage in patients without ARDS [22]. A sufficiently-powered randomized controlled trial investigating the effects of low tidal volume ventilation in patients without ARDS is presently lacking. PReVENT follows an earlier randomized controlled trial in The Netherlands [21] that was prematurely stopped because of an unexpected benefit (that is reduction in development of new ARDS). In contrast to the previous randomized controlled trial, PReVENT uses duration of ventilation as primary endpoint. This endpoint is objective and foremost clinically relevant. Namely, protracted use of ventilation is associated with serious physiological and psychological sequelae. Complications associated with ventilation are well-known and include VAP and ICU-acquired weakness. Psychological sequelae associated with protracted use of ventilation are post-traumatic stress disorder [38–40], anxiety and depression [41], delirium [30, 42] and cognitive deficits [43]. Also, from an economic perspective, any intervention that shortens duration of ventilation and/or ICU length of stay is highly relevant.

We anticipate PReVENT to be a highly feasible trial, as the study procedures are straightforward, without difficult or complex interventions. Furthermore, PReVENT uses a deferred consent strategy to include patients rapidly in the trial, and also those who are admitted during evenings or nights when researchers are frequently not around to ask for informed consent. This will create a study population representative of the average ICU population. A lean study protocol, not involving complex analyses and sampling of, for example, blood or lung lavage fluid, will limit the burden on daily activities.

PReVENT specifically prescribes a range of tidal volumes for each group. We aim to ventilate patients with tidal volumes of 4 ml/kg PBW or 10 ml/kg PBW, depending on randomization, but physicians and nurses are allowed to change the tidal volumes following strict guidelines: in case of a too high respiratory rate (in the lower tidal volume group), the tidal volume size can be increased stepwise to 6 ml/kg PBW - in case of too high airway pressures (in the higher tidal volume group), the tidal volume size can be decreased stepwise to 8 ml/kg PBW. Experiences with the study protocol suggest that the average tidal volumes will be 5 and 9 ml/kg PBW [21]. Notably, we aim for tidal volumes lower than 6 ml/kg PBW in the lower tidal volume group, since previous investigations suggest more benefit from further reductions in tidal volume size in patients with ARDS [10, 44–47]. Certainly, use of lower tidal volumes mandates use of higher respiratory rates to prevail adequate minute ventilation. Higher respiratory rates are typically seen as uncomfortable and maybe even unfeasible in non-sedated non-paralyzed patients. However, neither observational studies [48] nor the abovementioned randomized controlled trial in The Netherlands [21] suggested this to be a realistic problem. This was confirmed in a recently published meta-analysis that showed no differences in sedation needs between strategies using lower tidal volumes and those using higher tidal volumes [37].

At present, the size of tidal volumes in patients without ARDS is highly variable. For instance, two recent meta-analyses show that 11 % of patients are ventilated with tidal volumes of 4 to 6 ml/kg PBW and 53 % of patients are ventilated with tidal volumes > 8 ml/kg PBW [37, 49]*.* Also, the proposed level of PEEP is that level that is used as standard care in the participating hospitals, which is usually 5 cmH_2_O. The study protocol, however, advises on the best FiO_2_-PEEP-combinations [6].

From a physiological viewpoint one may argue that the seemingly ‘one size fits all’ approach that is used in this study does not allow individual titrations of tidal volumes. Indeed, more sophisticated approximations of the functional lung volume are possible: for example, by driving pressures as recently reported to be possibly more efficient in patients with ARDS [50]. It is highly uncertain, though, whether a similar relationship between driving pressure and outcome exists in patients without ARDS. Notably, the study protocol of PReVENT allows individual titrations, as tidal volume size could be adjusted depending on respiratory rate and airway pressures.

A patient’s height is necessary for calculating the size of tidal volumes to be used, as PBW is a function of patient’s height. Preferably we use height as reported in the medical record of each individual patient. If absent, for example, in patients who are acutely admitted from outside the hospital or when the medical record is not available, nurses and researchers must use tape measures. Nevertheless, measuring height in supine patients with a tape measure could result in height values slightly different from those obtained by measuring height in a standing patient [51]. This bias cannot be avoided.

In PReVENT patients will be provisionally included under a strategy of deferred consent. The reasons to choose for such a strategy are twofold: first, many if not all patients admitted for ventilation are incompetent to give informed consent; second, obtaining informed consent from a legal representative takes on average up to half a day [52]. Both experimental [53] and clinical studies [54–56] clearly show ventilation-induced lung injury is caused within hours. To keep the period of ‘uncontrolled’ ventilation as short as possible, using deferred informed consent, we can randomize patients within 1 hour of start of ventilation, or within 1 hour after arrival in the ICU.

Patients who present with ARDS according to the Berlin definition [25] are excluded from participation in PReVENT. In the first hour after intubation and start of ventilation, usually shortly after admission to the ICU, the medical history could be incomplete and the chest X-ray reading could not yet be available, leaving the attending physician only with the PaO_2_/FiO_2_ to exclude ARDS. Post-intubation atelectasis, usually short-lasting, cardiac failure and fluid overload, however, are the important causes for hypoxia in these patients. As patients with other reasons than ARDS for hypoxia should not be excluded from participation in PReVENT, we decided the following: in case the medical history is incomplete and the chest X-ray is not yet obtained, a PaO_2_/FiO_2_ between 200 and 300 mmHg is not a reason for excluding a patient from participation - patients with a PaO_2_/FiO_2_ < 200 mmHg, however, are not accepted, unless hypoxia is likely explained by cardiac failure or fluid overload. This approach is pragmatic but may not be perfect: it is possible that a small proportion of patients are diagnosed with ARDS in the following hours. Of course, then tidal volumes must be lowered to 6 ml/kg PBW - these patients, however, do not leave the trial, as the primary analysis follows the intention-to-treat principle. It is also possible that patients with a PaO_2_/FiO_2_ < 200 mmHg are excluded while not having ARDS. Here we prefer safety, and accept the risk of bias.

PReVENT knows several strengths. First, the study protocol is pragmatic and easy to follow. The trial is highly feasible, and inclusion of patients is not difficult. Also, PReVENT will be performed in the ICUs of different types of hospitals, increasing the generalizability of its findings. Furthermore, early contamination with the other ventilation strategy is prevented as much as possible by the rule that patients must be randomized to one of the two arms of the study within an hour.

One important limitation of PReVENT is that blinding is not possible due to the nature of the intervention, which could induce bias. However, the weaning process, which directly influences the primary outcome, stays within the hands of the attending ICU physician and nurse with no specific interest in PReVENT, and all analyses are performed in a blinded fashion. Second, sedation practice could mask potential patient discomfort caused by one or both ventilation strategies. However, it is stressed to aim for similar sedation aims in both arms of the trial. Third, when ventilation mode is switched from controlled - to spontaneous ventilation, tidal volumes may become less controllable and out of the predefined ranges. In a previous randomized controlled trial we experienced this to happen only within some hours after the switch; thereafter, tidal volume size was less difficult [21]. As with all multicenter studies, differences in care practice and complications such as ICU-acquired infections amongst the participating centers could be confounding factors. Also, we chose to accept a heterogeneous group of patients alike in a preceding trial of critically ill patients testing a similar intervention, and not to stratify for certain conditions [21]. If subgroups are large enough, we could decide to perform a *post-hoc* analysis.

In conclusion, PReVENT is the first national, investigator-initiated randomized controlled trial that is adequately powered to test the hypothesis that a ventilation strategy using low tidal volumes of 6 down to 4 ml/kg PBW benefits ICU patients without ARDS at onset of ventilation.

## Trial status

Currently recruiting.

## Abbreviations

APACHE II: Acute Physiology and Chronic Health Evaluation II
ARDS: Acute respiratory distress syndrome
BPS: Behavioral Pain Scale
CAM-ICU: Confusion Assessment Method for Intensive Care Unit
CCPOT: Critical Care Pain Observation Tool
CONSORT: Consolidated Standards of Reporting Trials
COPD: Chronic obstructive pulmonary disease
DSMB: Data and Safety Monitoring Board
ICU: Intensive Care Unit
LIS: Lung Injury Score
LOS: Length of stay
MRC: Medical Research Council
NRS: Numeric Rating Scale
PBW: Predicted body weight
PEEP: Positive end-expiratory pressure
RASS: Richmond Agitation Sedation Scale
SAPS II: Simplified Acute Physiology Score II
SDD: Selective decontamination of the digestive tract
SOFA: Sequential Organ Failure Assessment
VAP: Ventilator-associated pneumonia
VAS: Visual Analog Scale
VIDD: Ventilator-induced diaphragm dysfunction
VILI: Ventilator-induced
WMO: Medical Research Involving Human Subjects Act
